# Effects of Chinese medicines on monacolin K production and related genes transcription of *Monascus ruber* in red mold rice fermentation

**DOI:** 10.1002/fsn3.1511

**Published:** 2020-03-17

**Authors:** Lin Peng, Aisikaer Ai‐lati, Shuangping Liu, Zhongwei Ji, Jian Mao, Xin Che

**Affiliations:** ^1^ National Engineering Laboratory for Cereal Fermentation Technology Jiangnan University Wuxi China; ^2^ School of Food Science and Technology Jiangnan University Wuxi China; ^3^ National Engineering Research Center of Chinese Rice Wine Shaoxing China; ^4^ State Key Laboratory of Food Science & Technology Jiangnan University Wuxi China

**Keywords:** Chinese medicine, monacolin K, *Monascus ruber*, red mold rice, response surface methodology

## Abstract

Monacolin K (MK) is a secondary metabolite synthesized by polyketide synthases of *Monascus* spp. In this study, the combined supplementation of three medicines, including Citri Reticulatae Pericarpium (CRP), Fructus crataegi (FC), and *Radix Angelicae Dahuricae* (RAD), were mixed with nonglutinous rice and were optimized by response surface methodology to enhance the production of MK in fermented red mold rice (RMR). Under the optimum condition, MK production achieved 3.60 mg/g, which was 41.18% higher than RMR without medicines. The improved MK production was mainly caused by the up‐regulated transcription level of *mokA*, *mokB*, *mokF*, *mokH*, *mokI*, and *mplaeA*. Meanwhile, the inhibitory effect of *Poria cocos* (PC) on MK production (only 0.436 mg/g) was caused by significantly down‐regulated transcription of six tested genes. Therefore, this study is beneficial for better understanding of the possible mechanism of enhanced MK production by optimization of fermentation conditions.

## Graphical abstract

Mixed three Chinese traditional medicines enhanced monacolin K production in solid‐state fermentation of red mold rice. The possible mechanism may be attributed to the regulation of expression of genes related to MK synthesis.

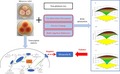

## 1 INTRODUCTION

Cardiovascular disease is the major factor leading mortality and morbidity of human, and hyperlipidemia is the main reason for its occurrence and development (Lewington et al., 2007). In 1979, Endo (1980) first isolated monacolin K (known as Lovastatin in *Aspergillus terreus* (Alberts et al., 1980)) from red mold rice (RMR) and found MK effectively inhibiting cholesterol synthesis. Currently, MK is used as hypocholesterolemic drug approved by FDA (Manzoni & Rollini, 2002). Due to its abundant content of MK, some RMR products are also used as anti‐hypercholesterolemic drugs, including LipoCol Forte, Cholestin, and Xuezhikang. Compared to tablets containing MK, the bioavailability of MK is significantly enhanced by oral administration of RMR products (Chen, Yang, Uang, & Lin, 2013). Besides lipid‐lowering effect, previous studies verified that MK also has several other activities, including preventing the formation of thrombus (Lee, Lee, Hwang, Lee, & Wang, 2013), reducing the occurrence of atherosclerosis (Lin, Li, & Lai, 2005; Wei et al., 2003), stimulating bone formation (Gutierrez et al., 2006), inducing the apoptosis of cancer cells (Kurokawa, Ito, & Matsui, 2017; Lee, Shih, Lee, et al., 2013), curing Parkinson's (Lin, Lin, Lin, Huang, & Lee, 2015) and Alzheimer's (Lee, Wang, & Pan, 2008) diseases. Therefore, RMR could be regarded as functional ingredient and applied in food industry.

Red mold rice is an important food additive used in some Chinese traditional foods and alcoholic beverages due to its abundant red pigments and MK. The function and color of red rice wine, a Chinese traditional alcoholic beverage, was caused by the addition of RMR during fermentation process (Zhou, 1996). With the increased attention paying to functional foods, some methods are developed to improve the production of MK in RMR, which could be used to further enhance MK content in foods. These methods include the optimization of culture medium (Panda, Javed, & Ali, 2010; Subhagar, Aravindan, & Viruthagiri, 2010; Suraiya et al., 2018) and the establishment of fermentation strategies in solid‐state fermentation (Gum, Nguyen, Lee, Han, & Cho, 2017; Lin, Wang, Li, Wu, & Chen, 2017; Tsukahara, Shinzato, Tamaki, Namihira, & Matsui, 2009). All these researches enhancing MK production in RMR were focusing on genes related to MK synthesis. After screening and characterization, nine genes related to MK synthesis in *Monascus pilosus* were identified by comparing with that of *A. terreus* genes related to lovastatin synthesis (Chen et al., 2008). Furthermore, the addition of some nutrients during RMR fermentation leads to the up‐regulation of some genes, which further enhance MK production (Huang, Liao, & Li, 2017; Zhang, Liang, Yang, Sun, & Wang, 2017). Therefore, the enhancement of MK production by optimization of culture medium is associated with the modulation of the expression of MK synthesis‐related genes.

In Chinese traditional medicines, some herbs could be used in both food processing and clinical treatment. Meanwhile, some functional components in Chinese medicines could effectively enhance the production of functional products in edible fungi (Zhou, Liu, Huang, Wu, & Yang, 2014). Therefore, we determined the effects of various Chinese medicines on MK production of *Monascus ruber* in RMR fermentation, and three Chinese medicines, including Citri Reticulatae Pericarpium (CRP), Fructus crataegi (FC), and *Radix Angelicae Dahuricae* (RAD), showed a significantly enhanced effect on MK production in *M. ruber* (Che et al., 2016). The addition amount of Chinese medicines was optimized by response surface methodology to obtain high MK production. Meanwhile, the transcriptional levels of six genes related to MK synthesis were investigated during RMR fermentation. The variety of transcription level between mixed medicines (enhanced MK production) and *Poria cocos* (PC) (inhibited MK production) was compared to gain better understanding of the modulation of genes related to MK synthesis.

## 2 MATERIALS AND METHODS

### 2.1 Microorganisms and solid‐state fermentation

*Monascus ruber* M2‐1 used in this study was isolated from commercial red mold rice and preserved in our laboratory (Che, Mao, Liu, Zhou, & Xue, 2016). The strain was incubated on potato dextrose agar medium containing 1.5% agar at 28°C for 7 days. Spore suspension preparation was prepared by adding sterilized water to *M. ruber* M2‐1 growing PDA plates and scraped aseptically. The concentration of spores in homogenous spore suspension was counted by hemocytometer. After dilution with sterilized water, 1.0 × 10^5^ spores/g rice were adding to the steamed nonglutinous rice mixed with various Chinese medicines, and moisture content was adjusted with lactic acid solution (pH 5.0) to 44%. Chinese medicines used in this study were Citri Reticulatae Pericarpium (CRP), Fructus crataegi (FC), *Folium mori* (FM), *Radix Angelicae Dahuricae* (RAD), and *P. cocos* (PC). In solid‐state fermentation, 35 g medium containing nonglutinous rice and Chinese medicines was placed in 250‐ml Erlenmeyer flask and incubated at 28°C for 12 days.

### 2.2 Experimental design and statistical analysis

In this study, Box–Behnken design was used to assess the main and interaction effects of various Chinese medicines: CRP (*x*_1_), FC (*x*_2_), and RAD (*x*_3_). The range and level of the variables analyzed in this study was shown in Table S1. The statistical model was based on the RSM by liner regression analyzing by software Design‐Expert version 8.4.

### 2.3 Biomass and moisture content analysis

Biomass during RMR fermentation was analyzed according to method reported by Liu, Xu, and Cen (2000). The intracellular nucleic acid of *M.* *ruber* mycelial in insoluble substrate (0.25 g) was extracted by 5% (v/v) trichloroacetic acid at 80°C for 30 min. After centrifugation (10,000 *g*, 4°C) for 15 min, the absorbance at 260 nm was measured. The standard curve was established from the value of OD 260 nm and biomass obtained in submerged fermentation, and this curve was used to calculate the biomass of *M.* *ruber* mycelial in RMR fermentation. The moisture of RMR during fermentation was analyzed by drying at 60°C for 12 hr.

### 2.4 Extraction and HPLC analysis of MK

Red mold rice (0.5 g) obtained from solid‐state fermentation was mixed with 30 ml 70% (v/v) ethanol, and MK was extracted by ultrasonic treatment (250 W, 40 kHz) at 50°C for 1 hr. After centrifugation (10,000 *g*, 4°C) for 5 min, supernatant was filtered by 0.22‐μm filter. The concentration of MK in supernatant was analyzed by HPLC under the following conditions: Athena C18 column (Athena 250 mm × 4.6 mm), mobile phase (acetonitrile:0.1% phosphorus acid = 65:35 v/v), flow rate 1.0 ml/min, column temperature 30°C, and wavelength 238 nm. The content was calculated according to MK standard (Sigma).

### 2.5 Quantitative real‐time PCR

Mycelial in red mold rice was frozen by liquid nitrogen, and total RNA was extracted by TRIzol Total RNA Purification Kit (Sangon), and reverse transcription was carried out with RevertAid First Strand cDNA Synthesis Kit (Fermentas). The 20 μl reaction mixture contained 2 μl cDNA as template, 1 μM primers, along with SYBR green reagent (TaKaRa) according to manufacturer's instruction. Primers for genes related to MK synthesis were listed in Table 1. The amplification program was described as follow: 94°C for 5 min, followed by 40 cycles of 94°C for 30 s, 54°C for 30 s, and 72°C for 2 min. Gene expression level was normalized against that of *GAPDH* gene expression.

**Table 1:** Primer sequences for genes related to MK synthesis

Gene	Sequence		Length (bp)
*mokA*	Forward	CCGTGAAACCTTGCTCTG	1,151
	Reverse	TCTGATGGGCTACGACTACA	
*mokB*	Forward	CATAGCTGTAGTGGGCA	1,658
	Reverse	TGCTCGTCGATATTCTCG	
*mokF*	Forward	CGCCCAATCCGAGACGTTAT	1,425
	Reverse	CGTTGGGTCGATTTGCTGTAATAG	
*mokI*	Forward	GAATGTATGGTCTATCCCTTTA	1,705
	Reverse	TTCCGTTTACGCTGTAGTG	
*mokH*	Forward	TTATACCCAGTCTTGGATGACTCGC	1,464
	Reverse	CTAGATTTCTCAGAACTAAATCTATCTATTTA	
*mplaeA*	Forward	AGGTGCGTTGGCCTGATGTT	1,194
	Reverse	GTGATTCAATTGGAAATTGGCTTC	
*GAPDH*	Forward	TTGAGGTCCACTATGCTGTA	1,013
	Reverse	GCGATGTAGGCAATGAGG	

## 3 RESULTS AND DISCUSSION

### 3.1 Effects of various Chinese medicine on MK production in solid‐state fermentation

**Figure 1:**
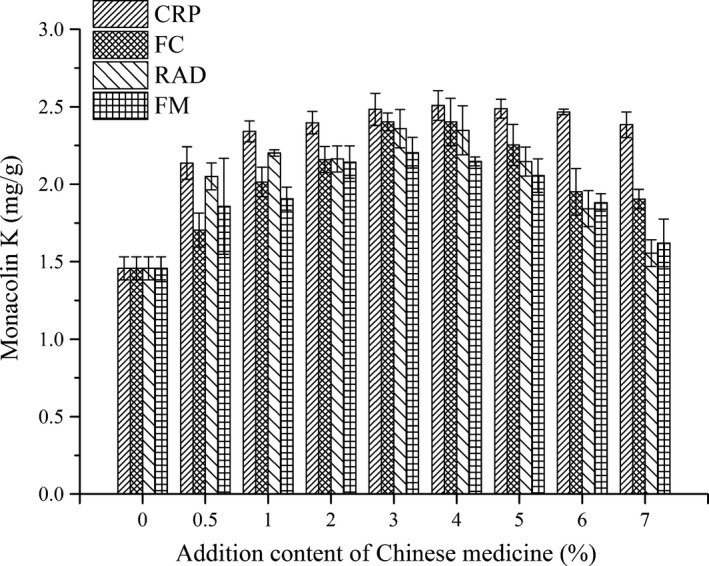
Monacolin K production by *Monascus ruber* with supplementation of different concentration of Citri Reticulatae Pericarpium (CRP), Fructus crataegi (FC), *Folium mori* (FM), and *Radix Angelicae Dahuricae* (RAD)

In our previous study, a MK‐producing strain *M. ruber* M2‐1 was isolated from commercial RMR, and 15 Chinese traditional medicines were mixed with nonglutinous rice to assess effects on MK production (Che et al., 2016). In these tested Chinese medicines, we found four medicine, Citri Reticulatae Pericarpium (CRP), Fructus crataegi (FC), *F. mori* (FM), and *Radix Angelicae Dahuricae* (RAD), effectively enhanced the MK production in 12‐day solid‐state fermentation. In this study, various additional amount (0.5–7 g/100 g) of four medicines were mixed with rice, and MK production in RMR was shown in Figure 1. Among these tested conditions, the highest MK production of 2.508 mg/g was achieved at an additional of 4 g/100 g CRP. Meanwhile, order preference of four medicines on MK production was also performed by (Technique for order preference by similarity to an ideal solution, TOPSIS), and results indicated that the preferred order was as follow: CRP, FC, RAD, and FM. Therefore, CRP, FC, and RAD were mixed to add into RMR fermentation, and optimized addition amounts of these three medicines for MK production were further determined by response surface methodology.

### 3.2 Response surface analysis

As shown in Table S2, MK production ranging from 2.890 mg/g to 3.550 mg/g was obtained under various conditions. Results were fitted into a second order quadratic model of coded units, and equation was listed as follow:

(1) $$y=3.53+0.076x_{1}+0.013x_{2}+0.034x_{3}-0.040x_{1}x_{2}-0.028x_{1}x_{3}-0.018x_{2}x_{3}+0.15x_{1}^{2}+0.19x_{2}^{2}+0.26x_{3}^{2}$$

In this obtained equation, MK production had linear and quadratic effects with three process variables. The correlation value (*R* ^2^) was .9865, indicating that 98.65% of the total variation in results can be attributed to the independent tested variables. Therefore, the value of Equation (1) had a good agreement between predicted and experimental MK production in this regression model. Statistical analysis based on ANOVA for model was shown in Table S3. The value of *p*‐value indicates that there is very low chance for *F*‐value occur due to noise in the experiments. Besides, the “Lack of Fit *F*‐value” of 13.01 implied that this value was not significant relative to pure error. Therefore, only a 7.22% chance for “Lack of Fit” occurring due to noise factor caused by experimental errors.

**Figure 2:**
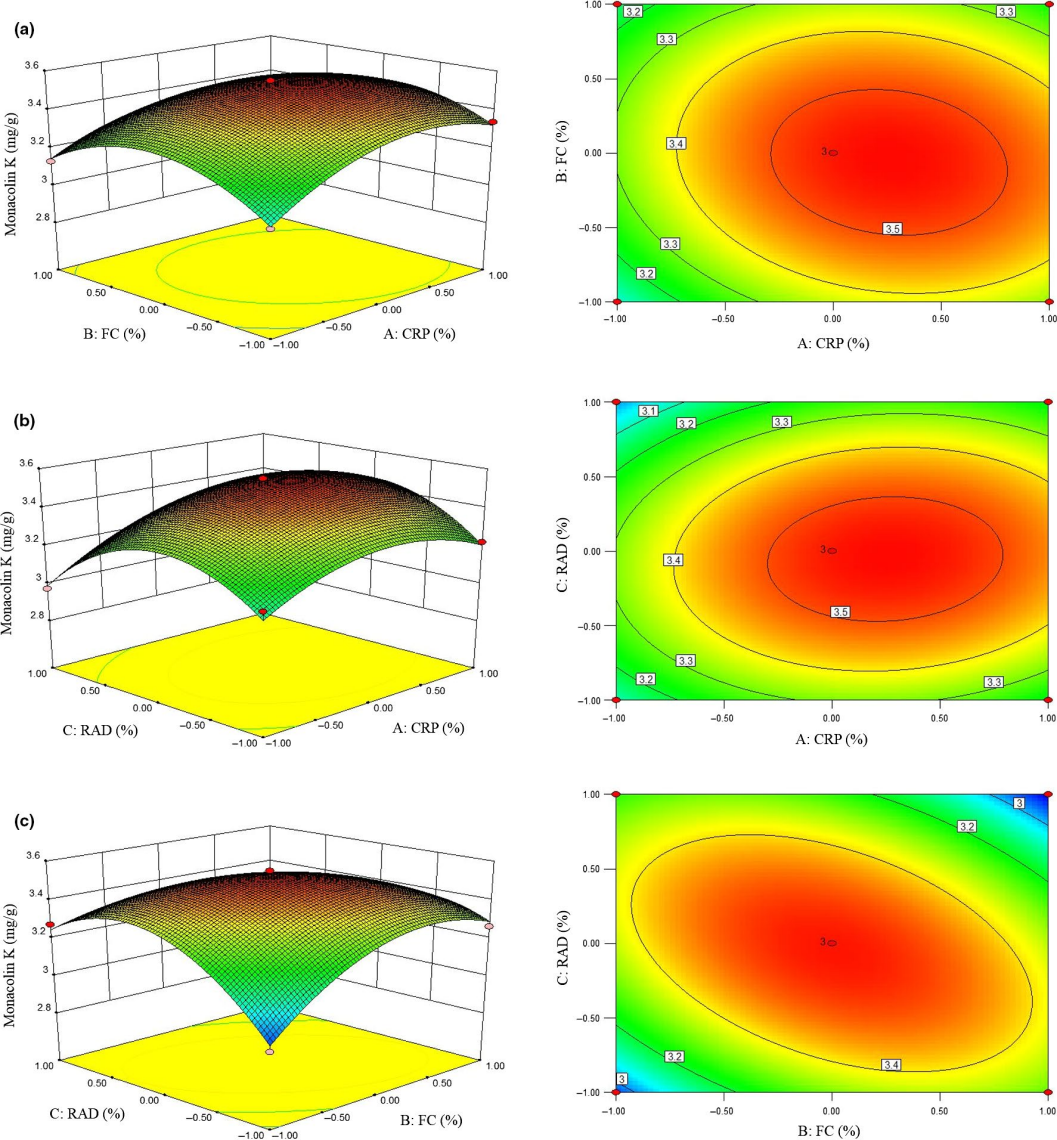
Response surface plots showing effects of three tested parameters on monacolin K production during solid‐state fermentation

In this study, the interactions between variables were investigated to show the significant effect on the MK production. Effects of CRP and FC addition into RMR on MK production were analyzed in three‐dimensional surface response and two‐dimensional interaction plots (Figure 2a). With the level increasing from −1 to 1, MK production of RMR added with CRP or FC showed an upward‐before‐downward trend. Similarly, MK production in interaction experiment of CRP and RAD (Figure 2b), or FC and RAD (Figure 2c), was also shown an upward‐before‐downward as addition level increasing from −1 to 1. After optimization by response surface methodology, the optimum combination conditions for maximum MK production was obtained and conditions were composed of: 3.75% CRP, 2.55% PC, and 2.01% RAD. Under this optimum condition, the predicted MK production was 3.545 mg/g. The optimum condition was further verified by three independent experiments, and the average experimental MK yield was 3.6 mg/g, indicating that the experimental value is well in agreement with the predicted value (Table S4). Meanwhile, a relatively insignificant error of 2.1% was also obtained from three independent experiments. It can be concluded that the proposed model is adequate for predicting the MK production in RMR fermentation with the addition of three Chinese medicines.

### 3.3 Comparison of MK production in RMS containing optimized medicines or *Poria cocos* (PC)

**Figure 3:**
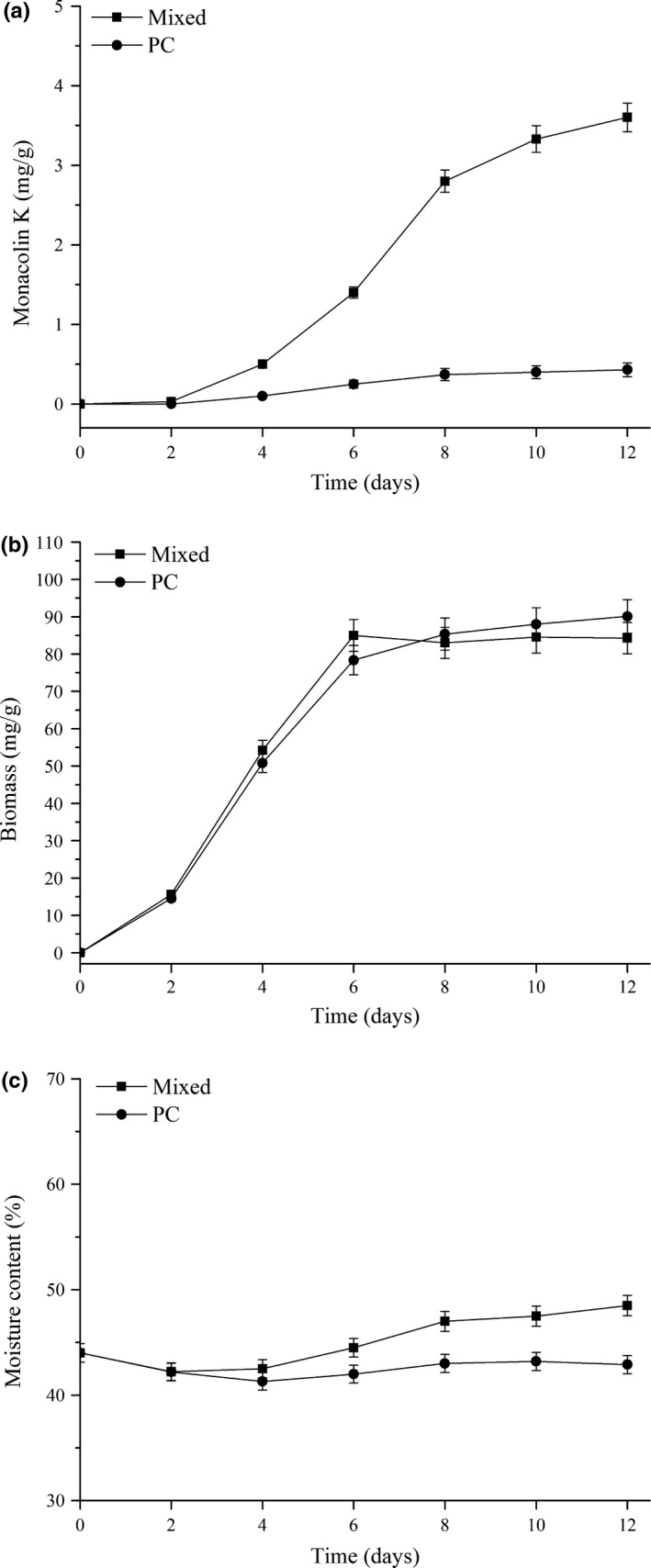
Monacolin K production (a), biomass (b), and moisture content (c) of red mold rice during solid‐state fermentation with supplementation of mixed medicines or *Poria cocos* (PC)

Among 15 medicines tested in previous study, we also found medicine *P. cocos* (PC) had negative effect on MK production, which was only 0.478 mg/g (Che et al., 2016). Therefore, the MK production, biomass, and moisture content of RMR containing optimized medicines or PC were analyzed to compare the difference during 12‐day fermentation (Figure 3a). Results showed that MK content in RMR with mixed medicines increased during the whole fermentation time, and the highest content was 3.601 mg/g on day 12. However, the MK production of RMR with addition of PC during time course was not changed, and only 0.431 mg/g MK was determined at the end of fermentation. The result indicated that MK production was obviously inhibited by the addition of PC, comparing with MK production (1.472 mg/g) in control without adding medicines (data not shown). Moreover, we found that biomass and moisture content of RMR with mixed medicines or PC had a similar curve during 12‐day solid‐state fermentation (Figure 3b,c). Therefore, effects of Chinese medicine (positive or negative) on MK production in RMR fermentation were not achieved by affecting fermentation environment.

### 3.4 The expression of genes related to MK biosynthesis

Monacolin K is a secondary metabolite produced by *Monascus* and shares the same structure with lovastatin produced by *A. terreus*. Chen et al. (2008) design the conserved region of *lovB* gene in *A. terreus* and identify nine gene (*mokA‐I*) related to MK synthesis in *M. pilosus*. All these identified gene in *M. pilosus* are highly homologous to genes related to lovastatin synthesis in *A. terreus* (Kennedy et al., 1999). The synthesis of monacolin K is directly associated with polyketide synthases (PKS). Lovastatin nonaketide synthase (LNKS) and lovastatin diketide synthase (LDKS), belonged to PKS, are responsible for the synthesis of nonaketide and diketide, which are substrate for MK synthesis. In *Monascus*, LNKS and LDKS are encoded by *mokA* and *mokB*, respectively, which share approximately 70% similarity (Chen et al., 2008). Moreover, trans‐esterase encoding gene *mokF* also modulates MK synthesis, and expression level of *mokF* is related to MK production (Zhang et al., 2017). Besides PKS and trans‐esterase, MK synthesis is regulated by transcription factor and some other regulators. The gene *mokH* is encoding transcription factor acting as the linker region of GAL4 and PPR1 and is regarded as the activator for MK production (Chen, Yuan, et al., 2013). Moreover, the methyltransferase gene (*laeA*) is regarded as a global regulator for secondary metabolites cluster of filamentous fungi (Lee, Lee, & Lee, 2013), and deletion of *laeA* blocks the expression of several secondary metabolite gene cluster (Bok & Keller, 2004). In *M. pilosus*, a similar methyltransferase gene (*mplaeA*) is found to have similar conserved region, and down‐regulation of *mplaeA* resulted in the decrease of MK production (Zhang & Miyake, 2009). Therefore, the gene *mokH* and *mplaeA* may regulate the MK synthesis in *M. ruber*. In addition, *mokI* shares 81% similarity to *lovI*, which acts as efflux pump in MK synthesis (Chen et al., 2008). Gutierrez et al. (2006) reported that the maximum MK content of intracellular in submerged fermentation is approximately fivefold higher than that of extra‐cellular, and this may be attributed to the block of *mokI* expression.

**Figure 4:**
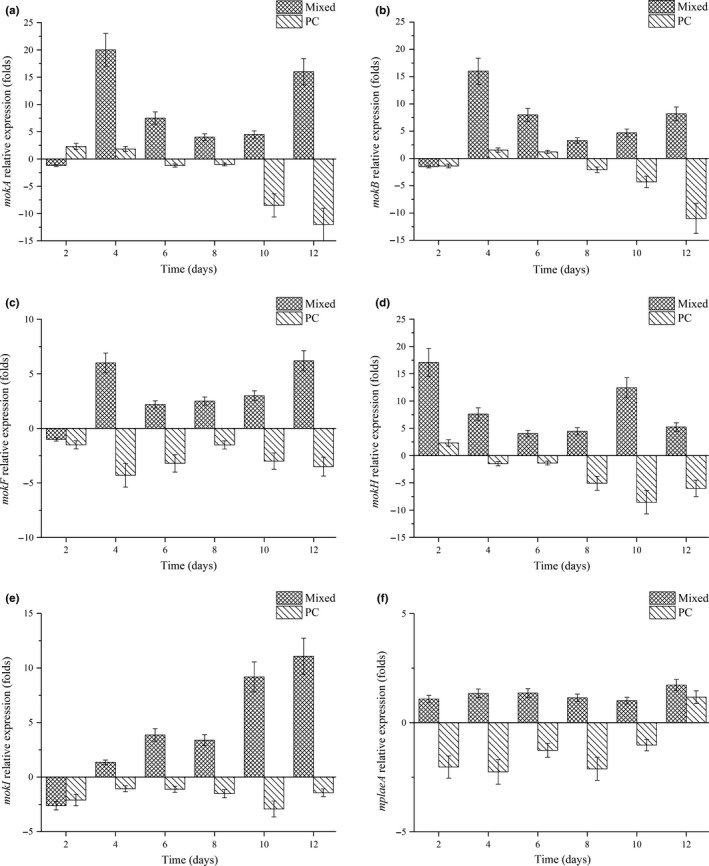
Effects of mixed Chinese medicines and *Poria cocos* (PC) on the transcription level of *mokA* (a), *mokB* (b), *mokF* (c), *mokH* (d), *mokI* (e), and *mplaeA* (f)

All genes discussed above were highly related to MK synthesis in *Monascus*. Therefore, the transcription level of *mokA*, *mokB*, *mokF*, *mokH*, *mokI,* and *mplaeA* was analyzed by qRT‐PCR. The function of these genes was investigated under RMR fermentation with optimized medicine condition (positive) or PC (negative), and results were shown in Figure 4. During 12‐day fermentation with PC, transcription of *mokA*, *mokB,* and *mokH* was positively detected only on the beginning of fermentation (day 1 to day 3), and then down‐regulated after day 6 (Figure 4a,b,d). Comparing with RMR containing mixed medicines, the transcription level of six detected genes was all highly down‐regulated during all fermentation time in RMR with PC (Figure 4). It has been reported that the *mokA*‐disrupted *M. pilosus* strain completely block MK production, indicating the importance of *mokA* gene in the MK synthesis (Chen et al., 2008). Meanwhile, high transcription levels of *mokE*, *mokF*, and *mokH* are effectively enhanced the production of MK in *Monascus* (Chen, Yuan, et al., 2013; Lin et al., 2018; Zhang et al., 2017). Therefore, the decrease of MK production after adding PC is achieved by the transcription suppression of genes related to MK synthesis and transportation. The accumulation of MK in RMR containing PC may be achieved at the beginning of fermentation, when the transcription of *mokA*, *mokB*, and *mokH* is not completely blocked.

**Figure 5:**
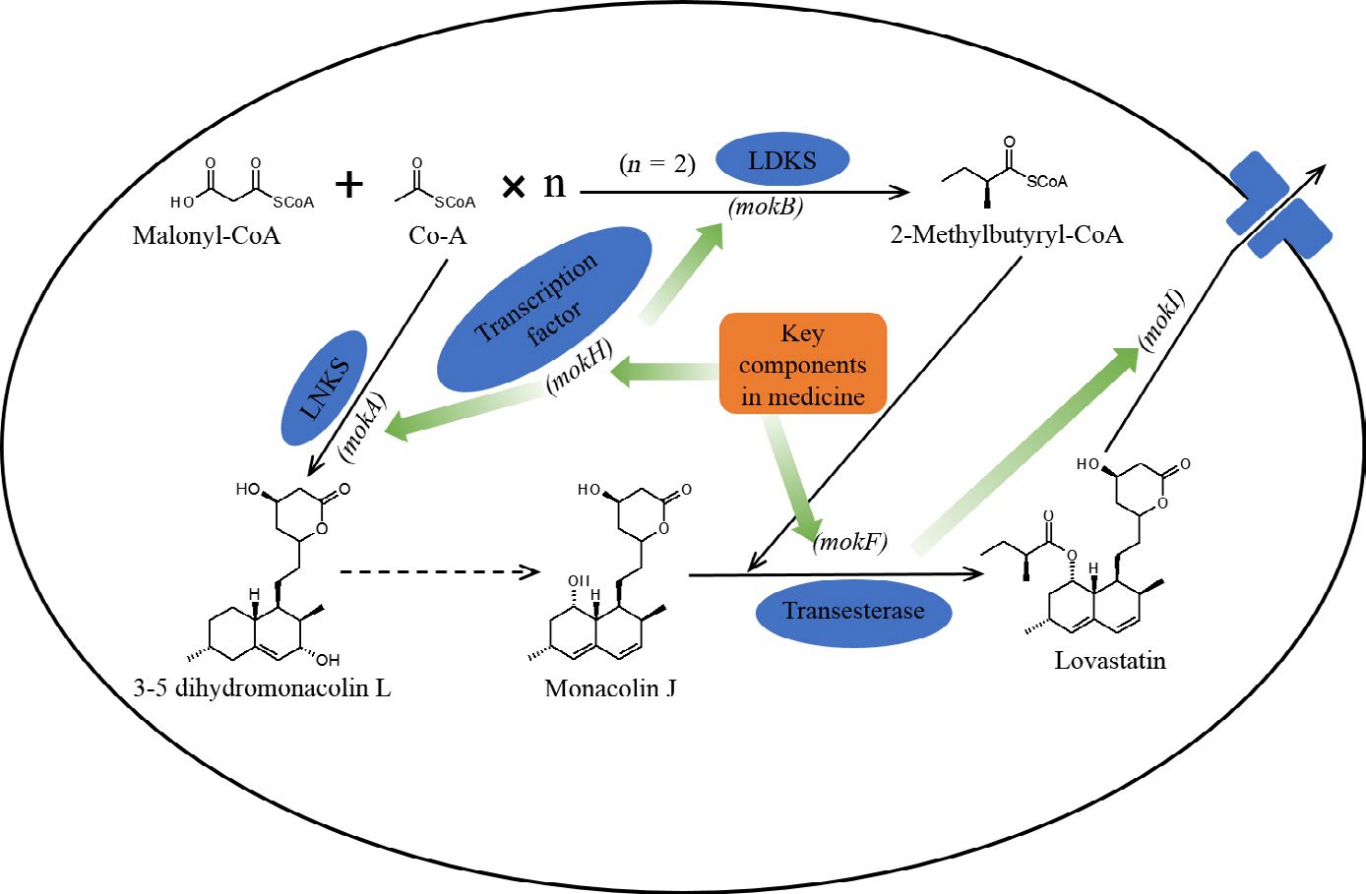
Possible mechanism of Chinese medicines modulating the transcription level of genes related to monacolin K production in *Monascus ruber*

Depending on this study, we proposed the possible biosynthesis pathway of MK production by *M. ruber* influenced by Chinese medicines (Figure 5). One or some key components in Chinese medicines are transported into intracellular of *M. ruber* mycelial and influence (down‐ or up‐regulate) the transcription level of PKS (LNKS and LDKS) and trans‐esterase, which affect the synthesis of precursors for MK. Meanwhile, key components also influence the expression transcription factor (encoded by *mokH*) of MK and regulator (encoded by *mplaeA*) of secondary metabolites, and these two regulators further affect the synthesis of precursors and MK. In addition, MK transportation protein encoding by gene *mokI* is also highly related to the secretion of MK. Further research focused on the identification of key components in Chinese medicine may be beneficial for better understanding the mechanism and pathway of MK synthesis in *Monascus*.

## 4 CONCLUSION

In this work, MK production of *M. ruber* in RMR fermentation was effectively enhanced with supplementation of combined three Chinese medicines. With the optimum combined addition of three medicines, MK production achieves 3.60 mg/g. Moreover, results of transcription level analysis indicate that six genes in *M. ruber* involve in MK biosynthesis.

## CONFLICT OF INTEREST

The authors have no conflict of interest relevant to this work.

## ETHICAL APPROVAL

Experiments in this study did not involve any human or animal subjects.

## Supporting information

Table S1‐S4
