# Supplementary material for: Effects of Chinese medicines on monacolin K production and related genes transcription of Monascus ruber in red mold rice fermentation
Source: Food Sci Nutr. 2020 Mar 17;8(4):2134–42. doi: 10.1002/fsn3.1511 (PMC7174227; doi:10.1002/fsn3.1511)
Supplement: Supplementary file 1 — Table S1‐S4 [file FSN3-8-2134-s001.docx]

**Supplementary materials**

Table S1 Three variables in coded and natural unit

| Variables with designate | Code | Coded value | | |
| --- | --- | --- | --- | --- |
|  |  | -1 | 0 | 1 |
| CRP (%) | x_1_ | 2.50 | 3.50 | 4.50 |
| FC (%) | x_2_ | 1.95 | 2.58 | 3.21 |
| RAD (%) | x_3_ | 1.62 | 2.03 | 2.44 |

Table S2 Experimental design matrix and the yields of MK

| Design point | Coded process variables | | | MK (mg/g) |
| --- | --- | --- | --- | --- |
|  | x_1_ (CRP) | x_2_ (FC) | x_3_ (RAD) |  |
| 1 | -1 | -1 | 0 | 3.079 |
| 2 | 1 | -1 | 0 | 3.338 |
| 3 | -1 | 1 | 0 | 3.130 |
| 4 | 1 | 1 | 0 | 3.227 |
| 5 | -1 | 0 | -1 | 3.153 |
| 6 | 1 | 0 | -1 | 3.222 |
| 7 | -1 | 0 | 1 | 2.973 |
| 8 | 1 | 0 | 1 | 3.153 |
| 9 | 0 | -1 | -1 | 2.922 |
| 10 | 0 | 1 | -1 | 3.264 |
| 11 | 0 | -1 | 1 | 3.273 |
| 12 | 0 | 1 | 1 | 2.890 |
| 13 | 0 | 0 | 0 | 3.550 |
| 14 | 0 | 0 | 0 | 3.532 |
| 15 | 0 | 0 | 0 | 3.522 |

Table S3 Anova for response surface quadratic model

| Source | Degree of freedom | Sum of square | Mean square | *F*-value | *p*-value |  |
| --- | --- | --- | --- | --- | --- | --- |
| Model | 9 | 0.6032 | 0.0670 | 40.5558 | 0.0004 | Significant |
| *x_1_* | 1 | 0.0458 | 0.0458 | 27.6841 | 0.0033 |  |
| *x_2_* | 1 | 0.0013 | 0.0013 | 0.7715 | 0.4199 |  |
| *x_3_* | 1 | 0.0092 | 0.0092 | 5.5957 | 0.0643 |  |
| *x_1_ x_2_* | 1 | 0.0066 | 0.0066 | 3.9699 | 0.1029 |  |
| *x_1_ x_3_* | 1 | 0.0031 | 0.0031 | 1.8638 | 0.2304 |  |
| *x_1_ x_2_* | 1 | 0.1314 | 0.1314 | 79.5109 | 0.0003 |  |
| *x_1_^2^* | 1 | 0.0849 | 0.0848 | 51.3347 | 0.0008 |  |
| *x_2_^2^* | 1 | 0.1327 | 0.1327 | 80.2987 | 0.0003 |  |
| *x_3_^2^* | 1 | 0.2455 | 0.2455 | 148.5205 | 0.0001 |  |
| Residual | 5 | 0.0083 | 0.0017 |  |  |  |
| Lack of fit | 3 | 0.0079 | 0.0026 | 13.0145 | 0.0722 | Not significant |
| Pure error | 2 | 0.0004 | 0.0002 |  |  |  |
| Cor. total | 14 | 0.6115 |  |  |  |  |

Table S4 MK production of three independent experiments to validate model adequacy

| Run | *x_1_* (%) | *x_2_* (%) | *x_3_* (%) | Predicted (mg/g) | Experimental (mg/g) |
| --- | --- | --- | --- | --- | --- |
| 1 | 3.75 | 2.55 | 2.01 | 3.545 | 3.62 |
| 2 | 3.75 | 2.55 | 2.01 | 3.545 | 3.57 |
| 3 | 3.75 | 2.55 | 2.01 | 3.545 | 3.61 |
